# Mitochondrial dynamics at the intersection of macrophage polarization and metabolism

**DOI:** 10.3389/fimmu.2025.1520814

**Published:** 2025-03-24

**Authors:** Pan Li, Zhengbo Fan, Yanlan Huang, Liang Luo, Xiaoyan Wu

**Affiliations:** ^1^ Department of Environment and Safety Engineering, Taiyuan Institute of Technology, Taiyuan, China; ^2^ People’s Government of Huangshui Town, Shizhu Tujia Autonomous County, Chongqing, China; ^3^ College of Veterinary Medicine, Southwest University, Chongqing, China; ^4^ National Engineering Research Center of Immunological Products, Department of Microbiology and Biochemical Pharmacy, College of Pharmacy and Laboratory Medicine, Third Military Medical University, Chongqing, China

**Keywords:** mitochondrial dynamics, fusion, fission, macrophage, metabolism

## Abstract

Macrophages are vital sentinels in innate immunity, and their functions cannot be performed without internal metabolic reprogramming. Mitochondrial dynamics, especially mitochondrial fusion and fission, contributes to the maintenance of mitochondrial homeostasis. The link between mitochondrial dynamics and macrophages in the past has focused on the immune function of macrophages. We innovatively summarize and propose a link between mitochondrial dynamics and macrophage metabolism. Among them, fusion-related FAM73b, MTCH2, SLP-2 (Stomatin-like protein 2), and mtSIRT, and fission-related Fis1 and MTP18 may be the link between mitochondrial dynamics and macrophage metabolism association. Furthermore, post-translational modifications (PTMs) of mtSIRT play prominent roles in mitochondrial dynamics-macrophage metabolism connection, such as deacetylates and hypersuccinylation. MicroRNAs such as miR-150, miR-15b, and miR-125b are also possible entry points. The metabolic reprogramming of macrophages through the regulation of mitochondrial dynamics helps improve their adaptability and resistance to adverse environments and provides therapeutic possibilities for various diseases.

## 1 Introduction

Macrophages are important sentinels in innate immunity. Classically activated macrophages and alternatively activated macrophages play roles predominantly in inflammatory processes and injury repair or parasitic diseases, respectively (1). It has been reported that *in vivo* M1-like and *in vitro* classical activated macrophages and *in vivo* M2-like and *in vitro* alternatively activated macrophage are not completely equivalent (2). Undoubtedly, the different polarization forms of macrophages affect the immune regulation, which in turn determines the prognosis of host in adverse environments. The fate of macrophages is interfered by a variety of factors. For example: (1) Multiple signaling pathways and factors affect macrophage polarization. For example, PI3K/AKT/Rac-1 and peroxisome proliferator-activated receptor-γ (PPAR-γ) are involved in macrophage anti-inflammatory (3), Janus kinase (JAK)-signal transducer and activator of transcription (STAT) 1, Adenosine 5’-monophosphate (AMP)-activated protein kinase (AMPK)/nuclear factor kappa-B (NF-κB) signal pathways are involved in macrophage inflammatory response (4–6). (2) Micro-environmental nutrients and metabolites regulate macrophage metabolism and thus affect its polarization. Such as serine by regulating glutathione (GSH) synthesis (7), and γ-aminobutyric acid (GABA) perturbing macrophage oxidative phosphorylation (OXPHOS) via succinate-flavin adenine dinucleotide (FAD)-lysine specific demethylase1 (LSD1) (8) are involved in the regulation of LPS or LPS+IFN-γ induced macrophages. A creatine-related metabolic pathway is involved in IL-4 induced macrophage polarization (9). Endothelial cells secrete lactate through glycolysis, and lactic acid, after ingestion by macrophages, affects the acetylation of specific molecules through p300/CBP pathway, increases endothelium permeability, and finally affects the course of disease by regulating the polarization of macrophages (10, 11). (3) In recent years, the role of epigenetic modifications in macrophage polarization has been emphasized, which is also significant for trained immunity (12, 13).

As we listed above, macrophages in different microenvironment have different requirements for nutrients and immunity. Mitochondria are important multifunctional organelles in macrophages, including the core energy and metabolism stations (14), cell division (15, 16), signaling platform (17) and other biological functions. Thus, mitochondria have a non-negligible impact on macrophage fate decision. Mitochondrial morphology involves a series of temporal and spatial mitochondrial processes (18, 19), including fission and fusion, as well as adaptive behaviors such as mitochondrial autophagy (termly named as “mitophagy”) and mitochondrial transport, which are closely related to the biological activities and are the adaptive strategies of mitochondria in different cellular microenvironments. Therefore, changes in morphology, quality and location of mitochondria bestow macrophages energy supplementation and/or immunomodulatory assistances.

Mitochondrial dynamics regulates macrophage immunity by affecting macrophage immunity-related signals and pathways, and their entanglement has been extensively reviewed elsewhere (14, 18, 20, 21). LPS-activated macrophages benefit fragmented mitochondria for its increased mitochondrial fission through Stat2 promoting dynamin-related protein 1 (DRP1) Ser616 phosphorylation (22). Subsequent mitochondrial remodeling, such as fragmentation, loose cristae structure, and reduced membrane potential [ΔΨ(m)], promotes their function to ROS production, which further facilitate the pro-inflammatory response of macrophages (22). Inhibition of pyruvate dehydrogenase kinase (PDHK) in macrophages can promote mitochondrial fusion and reduce inflammatory stress (23).

Reparative macrophages show the aggregation of elongated mitochondria and induce mitochondrial fusion (24). Interferon regulator 1 (IRF1), a transcription factor that coordinates the expression of multiple inflammatory genes, it plays a key role in the removal of damaged mitochondria, macrophage autophagy, and inflammation control (25). Studies have also shown that IRF1 plays a bidirectional role in regulating mitochondrial dynamics, responding to TLR-induced macrophage mitochondrial fusion or fission in macrophages via a cascade reaction with CHIP (a HSC70-interacting protein) monoubiquitination translocation or ubiquitination degradation (26). Apart from IRF1, extracellular signal-regulated kinase (ERK1/2) is probably another bond in the mitochondrial morphological network that adapt to the polarization of macrophage. For one part, ERK1/2 is related to LPS induced polarization in RAW264.7 cells and THP-1 macrophages (27, 28), or alternatively activated macrophages in patients with endometriosis or gastric cancer-liver metastasis (29, 30). For another, ERK1/2 probably participates in the inhibition of mitochondrial-dependent apoptosis (31) and facilitates mitochondrial biogenesis in virtue of promoting CREB phosphorylation and finally increasing PGC1α (PPARγ co-activator 1 alpha, a mitochondrial biogenesis regulator) (32). However, ERK1/2 is proved to induce fragmented mitochondria by phosphorylating DRP1 at S616 to induce mitochondrial fission (33, 34), and mediate MFN1 (Mitofusin 1) T562-phosphorylation (35) to decrease mitochondrial fusion. Reviewed by Sabouny R. and Shutt T. E. concluded protein kinase A (PKA), protein kinase B (AKT) and cyclic adenosine monophosphate (cAMP) work effectively in facilitating hyperfused mitochondria, while ERK1/2 and Ca^2+^ promote fragmented mitochondria (20). Unfortunately, this paper does not correlate these mitochondrial morphology regulating signaling molecules with the polarization state of macrophages, but we predict that mitochondrial dynamics have connection with macrophage immunity. More significantly, Xie et al. reviewed and speculated that mitochondrial dynamic network regulation (e.g., MFN1/2) probably participate in the release of IFN-β (type I interferons) and activation of the inflammasome NLRP3 in macrophages (21). In conclusion, mitochondrial dynamic networks regulating the immune responses of macrophages have been extensively summarized, so we will not dwell on it.

In addition to the immune-related factors and/or pathways mentioned above in geared to mitochondrial dynamics and macrophage polarization, macrophage immunity is closely associated with intracellular metabolism. As all accepted, the high plasticity of macrophages is reflected in OXPHOS and glycolysis dependent metabolic switch, which supports the determination of the polarization fate of macrophages under different stimulations (36). In IL-4 induced macrophages, OXPHOS is the dominant metabolic state, accompanied by a complete tricarboxylic acid cycle (TCA) cycle and enhanced electron transport chain (ETC), while LPS+IFN-γ activated macrophages prefer to use glycolysis (37). Recently, it was uncovered that mitochondrial dynamics can reflect different polarization phenotype of macrophages. Classically activated macrophages show glycolysis and discrete mitochondria, however, OXPHOS metabolically active macrophage has elongated mitochondria (38). Based on this mitochondrial dynamics tether to macrophage metabolism coincident with polarization states, mitochondrial subcellular imaging has potential to monitor metabolic progression of macrophages and ultimately instruct clinical treatment. The conclusion above depends on mitochondrial fluorescence confocal imaging and ImageJ macro tools to analyze its mass (foodprint), branching, network morphology and size and other traits (38). In addition to these intuitive and visual methods, the fusion and fission of mitochondria take place under the coordination of complex life activities in cells. For example, the guanosine triphosphatases (GTPase) proteins MFN1 and MFN2 are responsible for fusion, and DRP1 is responsible for fission (39). In addition, mitochondrial dynamics have been shown to induce metabolic reprogramming in skeletal muscle atrophy or liver tumors (40), suggesting that mitochondrial dynamics have significant impacts on the fate of body health. Whether there are deeper connections between mitochondrial dynamics and macrophage metabolism, we will discuss those in the following part.

## 2 Mitochondrial fission and macrophage metabolism

### 2.1 Mitochondrial fission event

As we discussed above, cytosolic protein DRP1 is the primary regulate factor during mitochondrial fission (39). The post-translational modification (PTM) of DRP1 has been extensively summarized and annotated (41), such as ubiquitination, phosphorylation, palmitoylation, O-GlcNAcylation (OGA), etc. Different PTM or the same type of modifications occurs at different sites may have completely opposite effects on the reconciliation of mitochondrial state. Therefore, it is of great significance to study PTM.

**Figure 1:**
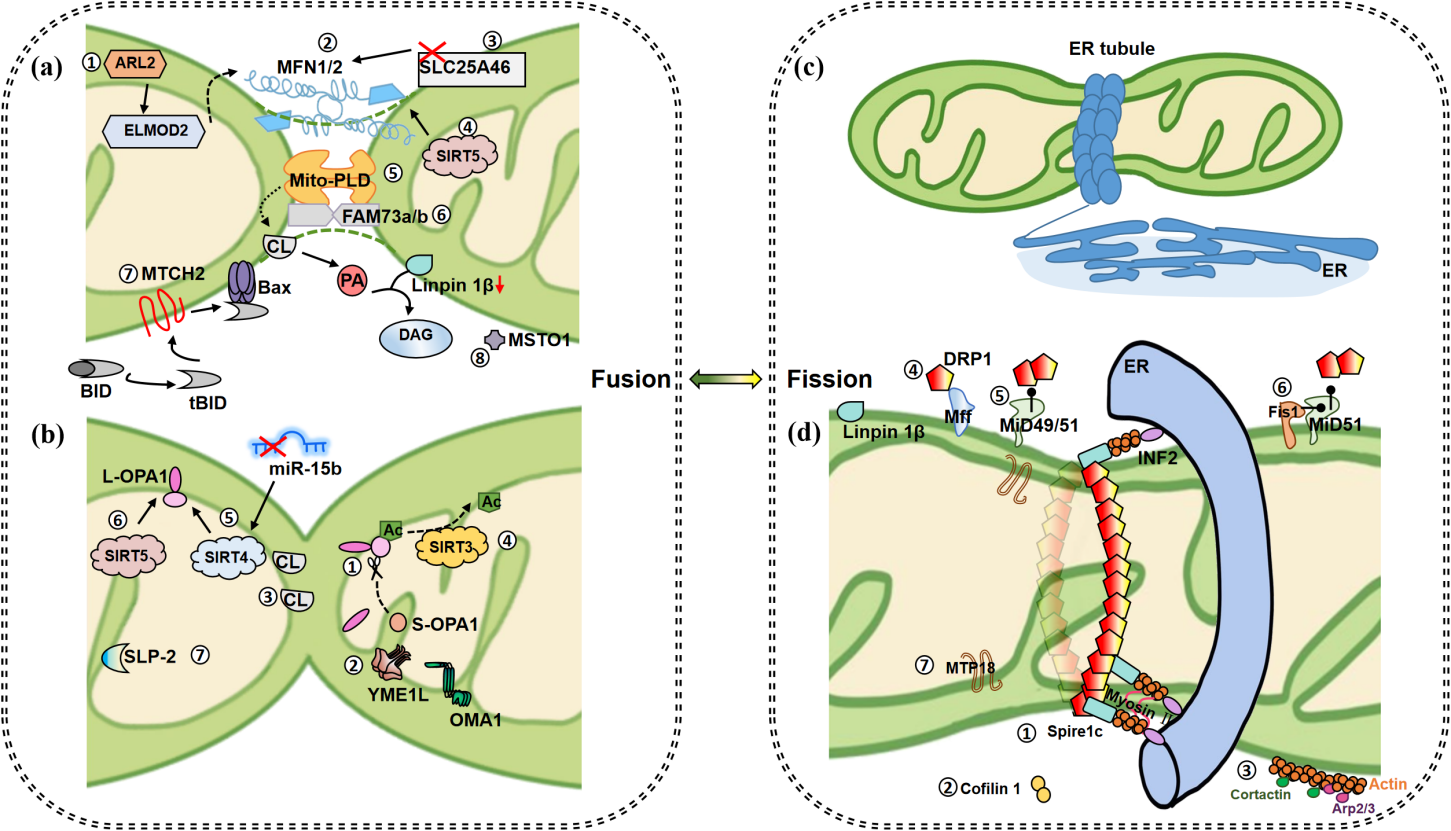
Mitochondrial fusion and fission events. **(a)** Fusion of OMM. ①IMS protein ARL2 promotes mitochondrial fusion during constitutive activation, and its downstream ELMOD2 is a necessary effector. ARL2 and ELMOD2 work upstream of MFNs. ②When MFN1/2 appear on two opposite membranes of mitochondria, it causes the fusion of OMM. ③Inhibition of OMM protein SLC25A46 promotes the stability of MFN1 and MNF2, which further promotes hyperfusion. ④SIRT5 overexpression promotes the increase of MFN2 and promotes fusion. ⑤At a certain time point when MFNs bind mitochondria together, MitoPLD modifies the lipid surface of the opposite mitochondrial OM in a PA-dependent manner for subsequent mitochondrial fusion. MitoPLD anchors on OM and hydrolyzes CL to produce PA. PA is converted into DAG by absorption of Lipin 1β, which ultimately contributes to mitochondrial fusion. ⑥Integrin protein FAM73A/B promotes the formation of mitoPLD dimer and inhibits its degradation. ⑦MTCH2 is a direct regulator of fusion, by regulating tBID and promoting the interaction between tBID and Bax, thereby inducing MOMP and accelerating apoptosis. ⑧MTSO1 is mainly located in the cytoplasm and is involved in OM fusion, but the specific mechanism is unknown. **(b)** Fusion of IMM. ①GTPase OPA1 contributes to IM fusion. Specifically, IMM fusion is dependent on L-OPA1 and its hydrolyzed form S-OPA1, and L-OPA1 alone is sufficient to promote mitochondrial fusion. ②OMA1 and YME1L are involved in the hydrolysis of L-OPA1 to produce c-, e- and d-type s-OPA1, respectively. ③IM fusion occurs asymmetrically. As long as OPA1 on one side and a sufficient concentration (15%-20%) of CL on the other side, these two elements bridge to promote fusion. S-OPA1 facilitates the bridging. ④SIRT3 promotes deacetylation of OPA1 at K926/931 to promote fusion. ⑤Up-regulated SIRT4 is accompanied by higher levels of L-OPA1 expression, a phenomenon that could also be repeated by inhibiting miR-15b. ⑥SIRT5 overexpression promotes the increase of OPA1, which contributes to mitochondrial fusion. ⑦SLP-2 cooperates with L-OPA1 to promote hyperfusion. **(c)** Initiation of mitochondrial fission. ER tubules wrap mitochondria to form early fission events. **(d)** Subsequent fission events. ①Located on the ER, INF2 recruits actin and forms a complex with Spire1c to promote mitochondrial contraction with the assistance of myosin II. ②The actin depolymerase protein cofilin1 acts as a reverse regulator to balance the effects of INF2/Spire1C. ③Cortactin and ARP2/3 complex regulate actin assembly on OMM and affect Drp1 dynamics and mitochondrial fission. Plentiful receptors on the OMM are involved in fission regulation. ④Drp1-Mff promotes GTPase-dependent shrinkage and breakage of fission helical rings. ⑤Drp1-MiD has been shown to promote MFN2 independent fusion by blocking Drp1. ⑥The activity of MiD51 is low when combined with Fis1, so Fis1 indirectly promotes fission. ⑦MTP18, located in the IMM, induces fission together with Drp1 and Fis1. Whether MTP18 plays a role in IM fission remains unknown. (Arrows represent promotion and dots represent inhibition.).

Mitochondrial fission is a multifactor derived biological event (**Figure 1**, right). Endoplasmic reticulum (ER)-Mitochondrial signaling plays a key role in mitochondrial fission, involving special regions of the ER called mitochondria-associated membranes (MAMs) (42). The ER tubule wraps mitochondrial tubule physically to form the earlier fission event (43), constitutes a subsequent assembly site and assists fission in an actin-dependent manner. In recent years, it has been found that acetylation of actin regulates actin polymerization via inverted guanidine protein 2 (INF2) located in the ER, and that mitochondria-localized myosin 19 (Myo19) collaborates with INF2 and spiretype actin nucleation factor 1 (Spire1C) to regulate actin assembly (44–46). Actin-depolymerizing protein cofilin1 acts as a reverse regulator to balance INF2/Spire1C in mitochondrial actin dynamics (47). Mff (the tail-anchored mitochondrial fission factor) controls midzone fission, leading to mitochondrial proliferation, while mitochondrial outer membrane protein fission 1 protein (Fis1) regulates peripheral fission, leading to mitochondrial autophagy (48). MiD49/51 (N-terminally anchored mitochondrial dynamics proteins, 49 kDa and 51 kDa) is responsible for Drp1 recruitment to the mitochondrial OM in the form of inactive dimer, and Drp1-MiD is essential for Drp1 assemble and subsequent cytochrome c (cyt c) release and cristae remolding during apoptosis (49). Mechanically, Hidenori et al. demonstrated visually through TEM that WT and Mff-KO cells showed abnormal mitochondria with balloon or lamella-less cristae, while most mitochondria in MiD49/51-KO and Drp1-KO cells retained lamellar cristae structure after apoptosis induction (49). This suggests that induced cristae stabilization in MiD49/51-KO and Drp1-KO cells leads to resistance to cyt c release (49). It was further demonstrated that MiD51’s N-SA, a mutation in the N-terminal OMM targeting signal anchor of MiD51, is necessary to correctly pinpoint the Drp1-MiD51 complex in the proximity of the crista-remodeling system under apoptotic signal, thereby modulating cristae junction disruption to release cyt c (49). In mammals, classical dynamin 2 (DYN2) assists DRP1 in promoting mitochondrial fission (40). In addition, cortactin, Arp2/3 (Actin-related protein 2/3) complex (50) and Myosin II also works in mitochondrial fission (51).

Little is known about mitochondrial IM fission events. Interestingly, mitochondrial protein 18 kDa (MTP18) induces fission along with Drp1 and Fis1 (52). Because located on IMM (mitochondria inner membrane), MTP18 may drive the IM fission assembly, which is only a speculation and needs experimental proof. In recent years, studies have shown that MTP18 deficiency can reduce mitochondrial division, and two microRNAs negatively regulate MTP18 (miR-652-3p and miR-668) (53).

In the ROS induced cardiac mitochondrial fission, MTP18 interfered with Drp1 accumulation but failed to mediate fission alone when Drp1 expression was scarcely (54). Therefore, MTP18 must collaborate with other factors to promote fission.

Mitochondrial fission is a complex biological process. We have summarized the current knowledge of fission-regulating proteins (**Table 1**), hoping to describe mitochondrial fission more comprehensively, but the existing content is far from sufficient. More research is still needed on mitochondrial fission, especially IMM.

**Table 1:** Mitochondrial dynamics related proteins and their location.

Protein	Homologs	Location	Effects in mitochondrial dynamics	References
DRP1	–	Cytoplasm	Fission↑	(55)
INF2	–	ER	Fission↑	(46, 56)
Spire1C	–	Mitochondria	Fission↑	(46, 56)
Cofilin1	–	Cytoplasm	actin cytoskeleton regulation, Fission↓	(47)
Mff	–	OMM	Fission↑	(57–59)
MiD49/MiD51	–	OMM	Fission↑	(49, 57, 58)
Cortactin	–	Cytoplasm	actin cytoskeleton regulation	(60)
Arp2/3 complex	–	Cytoplasm	actin cytoskeleton regulation	(60)
Myosin II	–	Cytoplasm		(60)
MTP18	–	IMM	Fission↑	(52)
Fis1	–	OMM	Fission↑	(52)
FUNDC1	–	MEM	Fission↑/↓	(61, 62)
MFN1/MFN2	Fzo	OMM	OM Fusion↑	(63, 64)
SLC25A46	Ugo1	OMM	OM Fusion↑	(65)
IMMT	Fcj1	IMM	cristae junctions↑	(65)
MTCH2	–	OMM	MOMP↑, apoptosis↑	(66–68)
MSTO1	Ftsz	Cytoplasm	Related to fusion	(69)
MtioPLD	–	OMM	OM Fusion↑	(70)
Lipin 1β	–	OMM	Fission↑	(71)
FAM73A/B	Miga	OMM	OM Fusion↑	(72)
OPA1	Mgm1	IMM	IM Fusion↑	(63, 73, 74)
OMA1	–	IMM	Cut L-OPA1 to S-OPA1 form c and e	(75)
YME1L	–	IMM	Cut L-OPA1 to S-OPA1 form d	(75)
ARL2	–	IMS	ARL2[Q70L]Fusion↑ ARL2[T30N]Fusion↓	(76)
ELMOD2	–	Mitochondrial matrix	Fusion↑	(77, 78)
SLP-2	–	IMM	Hyperfusion↑	(79)
SIRT3	–	Mitochondria	Fusion↑	(80, 81)
SIRT4	–	Mitochondria	Fusion↑ Fission↓	(82)
SIRT5	–	Mitochondria	Fusion↑	(83)

“↑” indicates increase and “↓” indicates decrease.

### 2.2 The connection between mitochondrial fission and macrophage metabolism

Preliminary studies of mitochondrial dynamics are usually performed in fibroblasts and/or stem cells (66, 84). Nowadays, mitochondrial dynamics have also been found to be crucial to the fate determination of macrophages. The classical cognition of macrophage metabolism is that glycolysis is the metabolic state of LPS-induced macrophages, and OXPHOS is extremely important for resting macrophages (85). The mitochondria of LPS (0.5 µg/mL) -induced BMDMs rapidly displayed small punctate and fragmented forms within 2 h, and remain in a fission state after 12 h of stimulation, and the expressions of *Mfn1*, *Mfn2*, *Fam73a*, and *Fam73b* are decreased, the dephosphorylation of Drp1 is enhanced (26). Mitochondria in IL-4-induced BMDM are tubular in fusion state, and *Fam73b* expression is elevated (26). Under the two completely different metabolic states of macrophages (glycolysis and OXPHOS), mitochondria also present extremely different morphology. Whether the dynamic changes of mitochondria can directly represent the metabolism of macrophages, or whether there are some ways to correlate these two phenotypes, we will analyze the argument in the following article.

**Figure 2:**
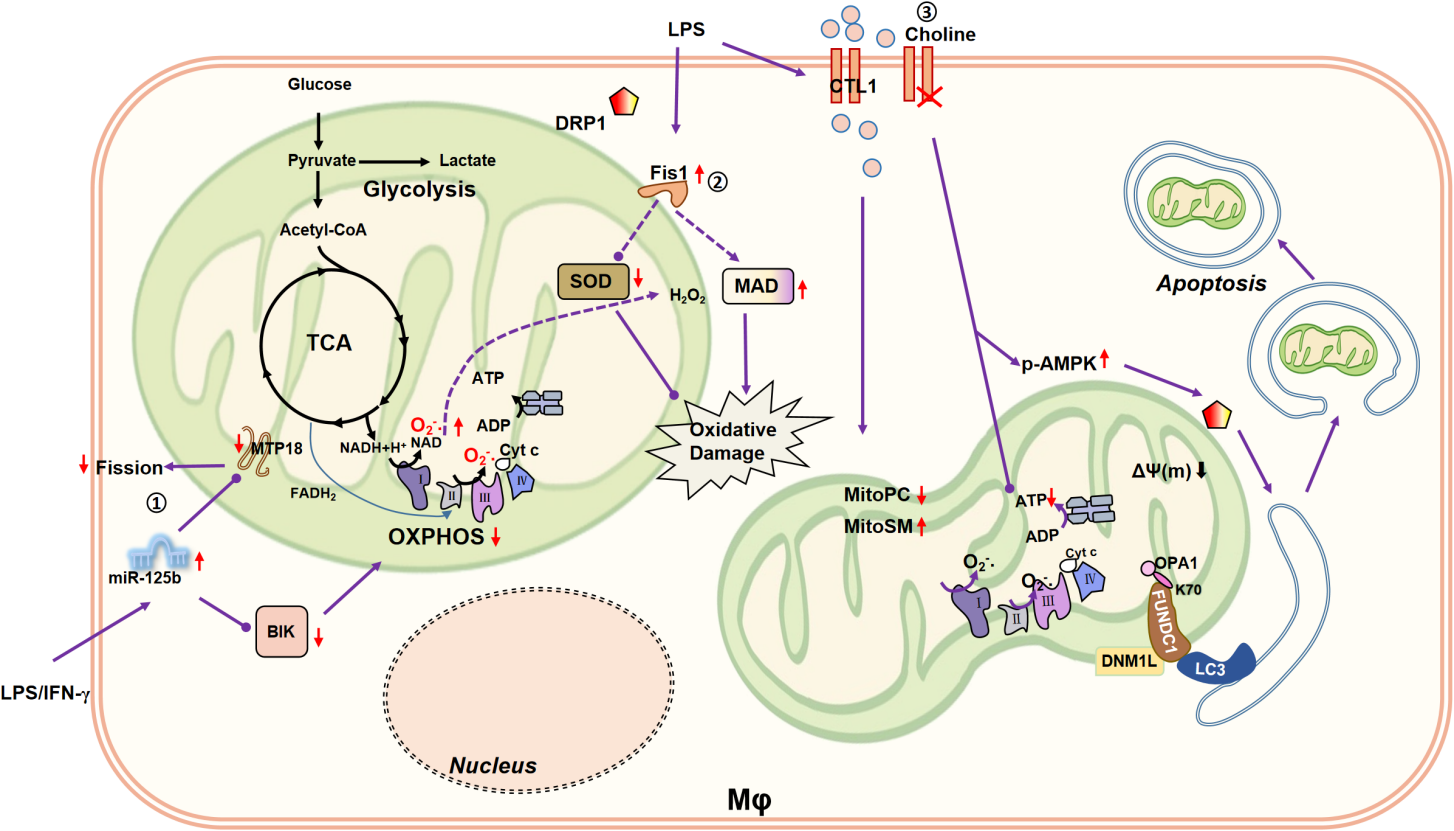
Connection between mitochondrial fission/mitophagy and macrophage metabolism. ①MiR-125b induces mitochondrial fusion by silencing MTP18 and reduces OXPHOS of macrophages by inhibiting BIK, reducing the pro-inflammatory activity of macrophages. ②Mitochondrial fission-related receptor Fis1 is upregulated in LPS (10 µg/mL)-induced rat alveolar macrophages, accompanied by a decrease in cellular SOD and an increase in MDA. SOD is capable to catalyze the disproportionation of O_2_•- to H_2_O_2_. Therefore, Fis1 may be involved in aggravated macrophage oxidative damage. ③LPS (0.2 µg/mL) enhances the expression of CTL1, promotes the choline uptake mediated by CTL1, and remodels macrophage lipid metabolism, such as reducing Mito-PC, and increasing Mito-SM. Impaired choline uptake disrupts mitochondrial ATP synthesis and triggers AMPK activation to promote DRP1-driven mitophagy.

In the process of mitochondrial fission, excessive activation of DRP1 leads to mitochondrial dysfunction, which is manifested as increased permeability of OM, decreased ATP production, and increased release of ROS and cyt c, thus leading to cell apoptosis (86). Therefore, mitochondrial fission is closely related to cell metabolism (**Figure 2**).

The crystal structure of MiD51, an adaptor protein of mitochondria, shows its ability to bind to ADP (87). MiD51 greatly promotes the hydrolytic activity and oligomerization of Drp1 in the presence of ADP, and then promoted mitochondrial fission, while when ADP absence, it inhibits the hydrolytic activity of Drp1 GTPase, which fully indicated the objective relationship between mitochondrial fission and cell metabolism (88, 89). MiD51 has been shown to regulate basal or glucose-induced oxygen consumption and insulin secretion levels in both mouse and human pancreatic islet cells (90). Unfortunately, there are few studies about MiD51 functions in macrophages at present, and the relationship between MiD51 and macrophage metabolism is also a study of far-reaching significance. Fis1, another key protein associated to mitochondrial fission, is up-regulated in LPS (10 µg/mL)-induced rat alveolar macrophage, and simultaneously causes cellular SOD (superoxide dismutase) decrease and MDA (malondialdehyde) increase, suggesting that Fis1 is involved in the aggravation of oxidative damage in macrophages (91). Whether the changes of SOD and MDA directly related to Fis1 was not explored in this article.

Recently, DRP1 has been confirmed to be involved in the differentiation of osteoclasts and the regulation of bone loss (92). It is well known that osteoclast differentiation involves a series of comprehensive metabolic reprogramming such as OXPHOS, glycolysis and fatty acid synthesis (93). Therefore, Drp1 regulation of osteoclast differentiation is closely related to macrophage metabolism regulation. However, it remains unclear whether there is a causal relationship between metabolic changes and mitochondrial dynamics. A large number of literature research to mitochondrial fission is associated with macrophage inflammatory state, including the mice BMDM and human monocytes THP-1 cells (22, 94, 95), but those research mainly focus on macrophage inflammatory signals, including inflammatory pathways or mediators, however, the metabolic changes of macrophages are worth exploring. In human monocytes, pro-apoptotic protein BIK and mitochondrial fission process 1 protein MTP18 correlate apoptosis with mitochondrial dynamics through the miR-125b (96). In monocyte derived macrophages, pro-inflammatory activity of macrophages are correlated with the decrease of BIK and MTP18 and the increase of miR-125b (96). Moreover, miR-125b induces mitochondrial fusion by silencing MTP18 and reduces the OXPHOS of macrophages (96).

Mitochondrial fission-related proteins have been studied more rarely on cell metabolism, let alone on macrophage metabolism (**Table 2**, **Figure 2**). Surprisingly, we find that micro-RNA (e.g., miR-125b) could be used as a breakthrough point to study the effect of mitochondrial fission on the metabolism of macrophages, which provides a good idea for subsequent research.

**Table 2:** The effects of mitochondrial dynamics related protein in cell metabolism.

Protein	Cell Type	Metabolic Changes	References
DRP1	HepG2 cells	ATP production↓, ROS↑, cyt c↑	(86)
MiD51	Pancreatic islet cells	Regulate basal or glucose-induced oxygen consumption and insulin secretion levels	(90)
Fis1	Rat alveolar macrophages	Cellular oxidative damage↑	(91)
MTP18	Human monocytes	Correlate with apoptosis	(96)
MiR-125b	Monocyte derived macrophages, THP-1	OXPHOS↓(Fusion↑)	(96)
FAM73b	BMDMs	Basal OXPHOS↑	(26)
MTCH2	BMSC, LPS stimulated RAW264.7	ROS↓,TCA↑	(84) (97)
YME1L cYKQ OMA1 active	Cardiomyocytes	Shift from lipid metabolism to the glucose metabolism	(98)
SLP-2	MEF	OXPHOS↑	(79)
SIRT3	Fibroblasts	OXPHOS↑	(80, 99)
	BMDM	OXPHOS↑	(100)
SIRT4	Fibroblasts	Mitochondrial respiration↓	(82)
	TAM	FAO↓	(101)
SIRT5	MDA-ME-231, C2C12	α-KG↓,TCA↓	(83)
	BMDM, PM	OXPHOS↑	(102)

“↑” indicates increase and “↓” indicates decrease.

## 3 Mitochondrial fusion and macrophage metabolism

### 3.1 Mitochondrial fusion event

Mitochondrial fusion largely divides into OMM fusion and IMM fusion (21, 103, 104). During mitochondrial fusion, the mixing of contents facilitates the transmission of information [e.g., mtDNA (105)] and leads to the rapidly dilution of cytoplasmic contents (106), which helps to rescue dysfunctional mitochondria. MFN1 and MFN2 are responsible for OMM fusion and optic atrophy 1 (OPA1) is responsible for IMM fusion (63). The fusion of two mitochondria occurs first in OM and then in IM (the inner membrane) (**Figure 1**, left).

#### 3.1.1 OMM fusion

About mitochondrial fusion and fission-related proteins were first discovered in yeast (107), drosophila (108), etc. Later, homologous proteins were found in humans and/or other mammals. *MFN1* and *MFN2* involved in OMM fusion in mammals are homologies of fuzzy onions (*Fzo*) (64). Similarly, Ugo1 is a OMM protein that coordinates the fusion events of OMM and IMM, but no obvious homologues of Ugo1 have been found in higher eukaryotes (109). The discovery of SLC25A46, a member of the mitochondrial solute carrier family 14 (SLC25), which matched Ugo1 in yeast (*Schizosaccharomyces japonicus*), confirmed that the OMM and IMM can fuse independently in mammalian cells (65). SLC25A46 may be recruited to the OMM as a pre-fission factor and interact with the IMM remodeling protein mitofilin (Fcj1), the IMMT homolog of mammal (65). Knocking down SLC25A46 causes delay in mitochondrial fission or elongation (65), reduced expression of SLC25A46 increases the stability of MFN1 and MFN2 (110), which both leading to mitochondrial hyperfusion. Interestingly, SLC25A46 mutation also results in hyperfusion of mitochondria, but with down-regulated OXPHOS, because excessive fusion seriously damages the subcellular structure of mitochondria, showing that the cristae structure is destroyed or even disappeared (111).

Mitochondrial carrier homolog 2 (MTCH2, SLC25A50), located at OMM, is a direct regulator of mitochondrial fusion/elongation in mouse embryonic fibroblasts (MEFs) and embryonic stem cells (ESCs) (66). As an effective binding partner of BID, MTCH2/MIMP (Met-induced mitochondrial protein) interacts with truncated BID (tBID) at OMM, promoting tBID translocation to mitochondria and ultimately inducing OMM permeabilization (MOMP) to accelerate apoptosis (67, 68).

Similar to Ftsz of prokaryotic (69), MSTO1 (Misato homolog 1), mainly located to the cytoplasm, is a protein involved in the fusion and network formation of OMM (69). Currently, MSTO1 is only known to be related to the fusion of OMM. It is worth exploring how MSTO1 participates in the fusion process, whether there is structural interaction with OMM, or whether MSTO1 promotes the mitochondrial fusion by enhancing the chemotaxis of other factors.

Intriguingly, remolding membrane phospholipid content may affect mitochondrial fusion. For example, mitochondrial phospholipase D (MitoPLD) belongs to Phospholipase D (PLD) signaling enzyme superfamily. A series of metabolic cascades catalyzed by MitoPLD results in mitochondrial tubular morphology, that is, promote mitochondrial fusion. Concretely, due to the special N-terminal domain, MitoPLD is anchored on OMM, which can hydrolyze cardiolipin (CL) on the surface of mitochondria to generate phospholipid acid (PA) (70). At certain points in time when MFNs tethered mitochondria together, MitoPLD modifies the lipid surface of the OM of the opposite mitochondria in a PA-dependent manner to perform the subsequent steps of mitochondrial fusion (70). Later studies found that PA absorption phosphatase (Lipin 1β) produced by MitoPLD hydrolysis converts PA into diacylglycerol (DAG) and terminates the lipid-signaling pathway of browning activated by PA (71). The catalytic domain of Lipin 1β locates at the tip of small mitochondria, or in a spot-like manner on tubular mitochondria, converting long mitochondrial tubules into medium-size fragments (71). Lipin 1β facilitates mitochondrial fission (71), and likewise, skeletal muscle of Lipin-1-deficient patients and extensor digitorum longus muscle of Lipin-1-deficient fatty liver dystrophy mice have been observed with mitochondrial aggregation or large shape (112–114). In conclusion, MitoPLD promotes mitochondrial fusion by lowering Lipin 1β through PA generation. On the other hand, PA generated by the MitoPLD is converted to DAG, which eventually leads to the increase of DAG. An animal study revealed that DAG probably reduces body fat mass by stimulating thermogenesis in brown adipose tissue (BAT) while inducing lipolysis in white adipose tissue (WAT) (115). From a more macroscopic perspective, MitoPLD shows that there is a correlation between cell metabolism and mitochondrial dynamics.

Moreover, mammalian *FAM73A* (*Miga1*) and *FAM73B* (*Miga2*) were discovered as orthologs of drosophila *mitoguardin* (*miga*) gene (72). As membrane integrins on OMM, MIGA1 and MIGA2 promote the formation of MitoPLD dimers and inhibit its degradation (72). Therefore, *miga* acts downstream of MitoPLD, and both *miga* and MitoPLD act downstream of *MFNs* to promote mitochondrial fusion. In addition to the effect on mitochondrial morphology, *miga* is also involved in the regulation of ΔΨ(m), mitochondrial ATP and ROS production (72). Therefore, *miga* may be a pleiotropic factor that affects mitochondrial morphological dynamics and cell metabolism.

#### 3.1.2 IMM fusion

*Mgm1*, the first member of the Dynamin family identified in yeast, is required for mitochondrial fusion of IMM and is also associated with the structure maintenance of crista (116), and the human homolog of *Mgm1* is *OPA1* (73). OPA1 is a dynamin-related GTPase that plays an important role in promoting fusion of IMM, maintaining cristae structure, promoting IMM structure and integrity (74) and controlling apoptosis (117, 118). OPA1 exists in two forms, namely, membrane-bound Long-OPA1 (L-OPA1) and Short-OPA1 (S-OPA1), the hydrolysate of L-OPA1 limited to the membrane space. Mitochondrial fusion is believed to depend on the presence of both L- and S-OPA1 (119), and various stress conditions destroy these complexes and trigger the complete transformation of L-OPA1 into S-OPA1, thus inhibiting mitochondrial fusion (119, 120). In recent years, it has been found that the L-OPA1 is sufficient to mediate mitochondrial fusion and has anti-apoptotic properties (121). Therefore, the morphology of OPA1 has a great influence on mitochondrial morphology.

The proteolytic sites S1 and S2 of OPA1 are encoded by exons 5 and 5B, respectively (122). The IM peptidase OMA1 and i-AAA protease YME1L cut OPA1 separately at S1 and S2, resulting in the aggregation or transformation of L-/S-OPA1 and interfering with mitochondrial fusion. The OMA1 cleaves L-OPA1 in S1 to generate S-OPA1 forms c and e (75). In *Oma1* knockout cells, fusion ability is preserved in the presence of L-OPA1, while the c and e forms of S-OPA1 are blocked, thus maintaining the tubular mitochondrial network and protecting cells from apoptosis (121). L-OPA1 protein in *Yme1l* knockout cells is equivalent to that in WT cells, but S-OPA1 accumulates due to OMA1 hydrolysis (121). This unbalanced processing of OPA1 results in the mitochondrial fragmentation, which also indicates that S-OPA1 may be involved in mitochondrial fission. According to the current conclusions, L-OPA1 is beneficial for promoting mitochondrial fusion, while S-OPA1 appears to be detrimental to mitochondrial fusion. However, S-OPA1, despite its lack of fusion function, has recently been shown to maintain cristae and mitochondrial energy through its GTPase activity (123), to some extent facilitating cell survival under stress.

Intriguingly, IM fusion has a different pattern from OM fusion. This is mainly reflected in the fact that OM fusion requires MFNs to appear on two opposite membranes, while for IM fusion, OPA1 regulates the fusion in an asymmetric way, which occurs as long as the unilateral IM contains OPA1 (124). Speaking of asymmetric regulation of OPA1, we have to mention a negatively charged acidic phospholipid CL. CL is enriched in IMM, and as long as there is sufficient concentration (15%-20%) on the other side of the mitochondria, L-OPA1 and CL pair to induce membrane-tethering and further induce mitochondrial fusion (125). S-OPA1 promotes the bridging between L-OPA1 and CL (125). However, fusion does not occur when the CL concentrations are low on the opposite or both sides of OPA1 (125). At this time, the interrelationship of homotypic *trans*-OPA1 mediates tethering of IM, which is conducive to the formation and maintenance of cristae (125). CL is re-assigned from IMM to OMM during the induction of damage signals such as mitochondrial damage and depolarization (126). In aging mitochondria, CL is oxidized by cyt c peroxidase and the content of CL decreases (127). Therefore, this regulatory mode of OPA1- CL^high^ bridging contributes to the specificity of mitochondrial fusion and may be involved in preventing the fusion of healthy mitochondria with damaged/senescent mitochondria and improving the efficiency of cells. However, the mechanism by which OPA1 combined with CL to regulate mitochondrial fusion remains unclear and needs to be explored experimentally.

In addition, CL is required for optimal activity of a variety of mitochondrial carrier proteins. For instance, CL endows ADP/ATP carrier (AAC) 2 with optimal activity by promoting the stable association between AAC2 and respiratory supercomplexs, and reduces the travel distance of cyt c between ETC complex III and IV, contributing to the efficiency of OXPHOS (128). AACs are responsible for the exchange of ADP/ATP, specifically the transfer of ATP formed by OXPHOS from IMM to the intermembrane space (IMS) (129). In addition to regulating ATP production (coupled energy conversion) in mitochondria, AAC is shown to mediate the uncoupled energy conversion (130). Therefore, in the process of regulating mitochondrial fusion with OPA1, CL is likely to associate mitochondrial dynamics with cell metabolism by influencing AACs.

#### 3.1.3 IMS proteins participated fusion

ARL2 (ADP-ribosylation factor (ARF) like 2) exists in the IMS and is uniformly distributed along the mitochondria in a spot-like pattern with MFN1/2 (76). The dominant negative mutation of ARL2[T30N] results in a decrease in mitochondrial fusion rate (24h) and a loss of specific mitochondrial motility (30h), and the dominant activating mutant ARL2[Q70L] leads to a significant increase in mitochondrial tubular structure (48h), independent of its effect on microtubules (76). Although there are some differences in the dynamics of cellular effects, as indicated activation time in parentheses, ARL2 generally promotes mitochondrial fusion during constitutively activation, and its reversal of mitochondrial fragmentation in the absence of fusion factors requires the presence of OPA1 and at least one MFN (76).

Richard A. et al. conducted the above systematic studies on ARL2 also found ELMOD2 (ELMO domain containing 2), which is essential for ARL2 to exert its active functions (131). ELMOD2 is an ARL2 GTPase-activating protein (GAP) that acts downstream of ARL2, and its influence on mitochondrial morphology is independent of GAP activity (77). In MEFs with MFN1 or MFN2 deficiency, the effect of ELMOD2 on the reverse of mitochondrial fragmentation is to a lower degree than that of ARL2, and ELMOD2 and ARL2 have no significant effect in MFN1 and MFN2 double-knockout cells (78). This also suggests that ARL2 and ELMOD2 both act upstream of MFNs, and ARL2 activation without ELMOD2 cannot induce mitochondrial elongation, so ELMOD2 is an effector necessary for ARL2 to promote mitochondrial elongation and tubular structure maintenance (77). Interestingly, ELMOD2 and ARL2, as well as MFN1, MFN2, and MitoPLD, are all located in a regular spot-like discrete distribution along the mitochondria forming bands (77), suggesting that they may form some special complexes in the mitochondria to function together, or there are unknown binding domains on the mitochondria, which recruits these factors to alter mitochondrial dynamics.

In the process of mitochondrial fusion, various elements of OM, IM, and IMS work together to change the dynamics of mitochondria, forming a complex and precise regulatory network (**Table 1**.). Among them, CL and DAG are involved in the metabolic regulation of OXPHOS and lipid metabolism, respectively, suggesting that mitochondrial dynamics is related to cell metabolism.

### 3.2 The association between mitochondrial fusion and macrophage metabolism

**Figure 3:**
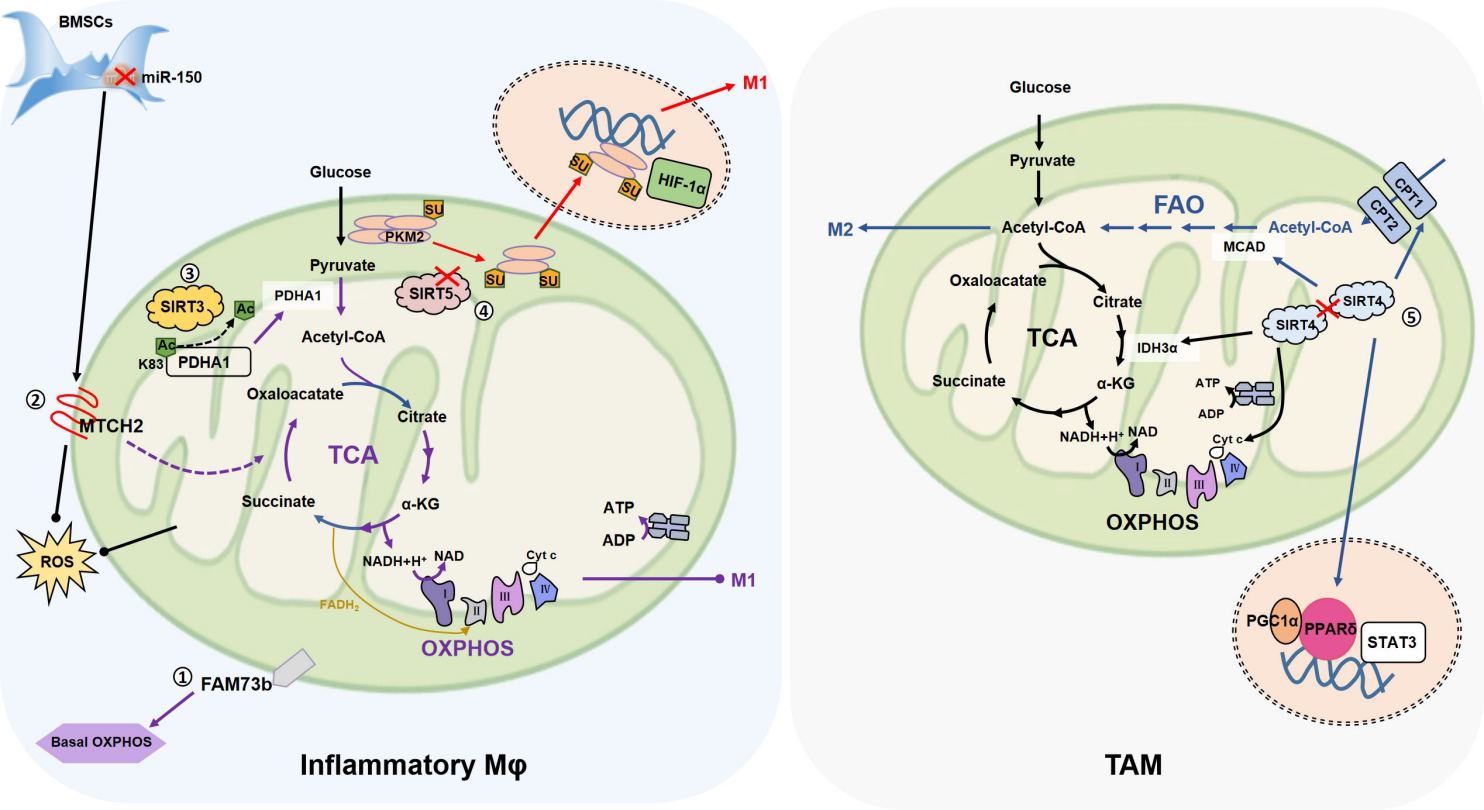
The association of mitochondrial fusion with macrophage metabolism. ①FAM73b which promotes mitochondrial OM fusion, promotes OXPHOS of resting macrophages, but has no effect on glycolysis, and neither OXPHOS nor glycolysis in inflammatory state. ②In the co-culture of BMSCs and macrophages, inhibition of miR-150 increases the expression of OMM fusion-related MTCH2 and promotes the transfer of mitochondria from BMSCs to macrophages (not shown in the figure). MTCH2 may enhance OXPHOS by promoting TCA cycle, thereby alleviating LPS (0.5 μg/mL)-induced apoptosis. ③MtSIRT promotes mitochondrial fusion, in which SIRT3 promotes PDHA1 deacetylation at the K83 site, and activated PDHA1 catalyzes pyruvate production of acetyl-coA to promote OXPHOS, inhibit glycolysis, and reduce macrophage inflammation. ④SIRT5 knockdown promotes PKM2 hypersuccinylation, reduces its pyruvate kinase activity, then induces PKM2 entry into the nucleus, and further persuading macrophage inflammation. ⑤SIRT4 knockdown activates FAO by promoting the expression of lipid catabolism genes such as MCAD and CPT1, and promotes the M2-like transformation of TAM through FAO-PPARδ-STAT3. On the other hand, SIRT4 silencing increases the expression of IDH3α, cyt c and other mitochondrial genes, so SIRT4 silencing contributes to the strengthening of mitochondrial OXPHOS. (Arrows represent promotion and dots represent inhibition.).

As we can see in **Figure 1**, a large number of proteins play a role in mitochondrial fusion and together maintain this complex biological process. Among them, no matter the OM proteins FAM73b, MTCH2, or mtROS are found to be involved in macrophage metabolism, while the relationship between IM proteins involved in fusion and macrophage metabolism still needs to be studied (**Figure 3**).

#### 3.2.1 FAM73b

Compared with wild type (WT) cells, FAM73b KO BMDMs show a severe decline in the basal OXPHOS level and the mitochondria are in a state of fragmentation (26). However, there is almost no difference in ECAR between WT and FAM73b KO BMDMs after LPS (0.5 µg/mL) stimulation and the difference in OCR are converged (26). These results suggest that FAM73b KO has little effect on the glycolysis ability of macrophages, and affects OXPHOS mainly in the resting state. Therefore, we speculate that mitochondrial dynamics affect macrophage metabolism, but are biased and depend on specific cell state.

#### 3.2.2 MTCH2

In bone marrow mesenchymal stem cells (BMSCs), inhibition of miR (microRNA)-150 mitigates LPS (0.5 µg/mL)-induced apoptosis in RAW264.7 (84), suggesting that the metabolic status of RAW264.7 is changed. The co-culture of BMSC also reduces ROS production of RAW264.7 after LPS stimulation (84). Mitochondrial respiratory chain is the main source of intracellular ROS, and the antioxidant system of mitochondria is responsible for removing ROS (132), so mitochondria play an important role in this process. As expected, the inhibition of miR-150 leads to the expression of MTCH2 (84), which is related to mitochondrial fusion (66). In addition, it is indicated that increased MTCH2 promotes mitochondrial transfer from BMSCs to RAW264.7 (84), so miR-150 may regulate the metabolism of RAW264.7 by affecting the morphology of transferred mitochondria. Unfortunately, this research did not cover studies related to mitochondrial dynamics, so this conclusion needs to be validated experimentally. On the one hand, miR-150 has been shown to negatively regulate brown-like transformation of white adipose tissue in inguinal peritoneum of mice, so regulates metabolism (133). In AML (acute myelocytic leukemia) cells, MTCH2 deletion has been shown to reduce glucose entry into the mitochondrial TCA cycle, thereby inducing glutamine to maintain the TCA cycle in the form of oxaloacetate, as well as reduced mitochondrial pyruvate levels and increased nuclear pyruvate and pyruvate dehydrogenase (PDH) levels (97). Further, in the cell nucleus, PDH converts pyruvate into acetyl-CoA and increases histone acetylation. This series of metabolic changes promotes the differentiation of AML (97). Therefore, we hypothesized that MTCH2, which affected mitochondrial fusion, is transferred from BMSCs to RAW264.7, which may promote TCA circulation by affecting macrophage metabolism, thus enhancing OXPHOS and alleviating LPS-induced apoptosis.

#### 3.2.3 OMA1/YME1L

Cardiomyocytes-specific deletion of *Yme1l* (cYKO) in mice inhibits the formation of S-OPA1 form d, and activated OMA1 accelerates the proteolysis of OPA1, leading to the accumulation of S-OPA1 form c and e, triggering mitochondrial fragmentation and leading to dilated cardiomyopathy and heart failure (98). The cYKO shifts the cardiomyocytes from lipid metabolism to the glucose metabolism, and this metabolic shift leads to heart failure in cYKO mice (98). However, double knockout mice (additional *OMA1*-deficient in *Yme1l* ^-/-^ mice) show normal cardiac function by preventing OPA1 cleavage (98). The additional muscle specific-knockout of *Yme1l* maintains cardiac function without restoring the mitochondrial morphological defects of cYKO cardiomyocytes (98). Interestingly, the loss of *Yme1l* in skeletal muscle systematically impairs glucose homeostasis, inhibits insulin secretion, and thus reduces cardiac glucose uptake, alters cardiac metabolism, and slows down cellular involuntary metabolism, demonstrating the physiological importance of tissue crossover disturbance (98). Metabolic intervention in cYKO mice fed a high-fat diet replicates the protective effect of hmYKO (heart and muscle-specific deletion of *Yme1l*) mice (98). In conclusion, coordinated mitochondrial dynamics or balanced mitochondrial fusion and fission plays a key role in cardiac metabolism and cardiac function. YME1L and OMA1 are still lacking in research in macrophages, but they affect mitochondrial dynamics by shearing L-OPA1 to produce different forms of S-OPA1, which may affect macrophage metabolism in this process, which also provides new ideas for studying the macrophage metabolism regulation.

#### 3.2.4 SLP-2

When mitochondria are exposed to selective stress in cells, mitochondrial hyperfusion occurs prior to mitochondrial fission (79). In this process, mitochondrial IM protein SLP-2 cooperates with L-OPA1 and MFN1 to induce hyperfusion, which in turn promotes the production of OXPHOS and ATP (79).

#### 3.2.5 MtSIRT

Mitochondrial Sirtuins (mtSIRT), including SIRT3, SIRT4 and SIRT5, are important parts of the sirtuin family (SIRT1-7) and possess enzyme activity such as NAD^+^-dependent deacylases and ADP-ribotransferase, which endowing mtSIRT with the function of regulating energy metabolism (134).

#### 3.2.6 SIRT3

On the one hand, SIRT3 promotes mitochondrial function by regulating the activity of metabolic enzymes, such as by binding to NDUFA9 subunits of ETC complexes I and deacetylation to maintain OXPHOS (99). SIRT3, on the other hand, directly binds to OPA1 at its lysine site (K926 and/or K931) for deacetylation, and enhances mitochondrial respiratory complex activity (80). Since mitochondrial morphology depends on the integrity of the coiled-coil domain at C-terminal of OPA1 (81), where these two acetylated lysine residues of OPA1 located, therefore, SIRT3 deacetylation promotes mitochondrial fusion (80). According to the TEM results, in SIRT3-KO heart, mitochondria are mostly clustered together, forming multiple island-like shapes, and the OMM is continuous, but the IMM fusion is defective (80), which also is consist with the function of OPA1 responsible for IMM fusion and reminds us that OMM fusion can occur independently of IMM fusion.

#### 3.2.7 SIRT4

Down-regulation of SIRT4 in aging chondrocytes was accompanied by excessive fragmentation of mitochondrial network, elevated ROS levels, and impaired ΔΨ(m) (135). Overexpression of SIRT4 saved mitochondrial fragmentation and restored mitochondrial homeostasis in aging chondrocytes (135). Therefore, SIRT4 has the potential to promote mitochondrial fusion. In addition to reported dehydrogenase activity (136, 137), in fibroblast models, SIRT4 expression inhibits mitochondrial respiration under basal conditions, resulting in reduced electron transport system capacity (ETS) and ΔΨ(m) (82). The up-regulated SIRT4 is accompanied by a higher level of mitochondrial fusion regulatory factor L-OPA1 expression, resulting in a higher degree of mitochondrial elongation/fusion and inhibiting mitochondrial fission and autophagy (82), and this phenomenon can also be repeated by upregulation of SIRT4 by inhibiting miR-15b (82). Meanwhile, the overexpression of SIRT4 in Muller glial cells increased the L-OPA/S-OPA1 ratio and MFN2 (138); SIRT4 positively regulates OPA1 and MFN1 in skeletal muscle (139); In mammary epithelial cells, SIRT4 deletion reduces MFN1/2 and increases DRP1 and Fis1 (140). Taken together, these evidences support the role of SIRT4 in promoting mitochondrial fusion.

#### 3.2.8 SIRT5

SIRT5 has deacetylase, deglutarylase and desuccinylase activities and participate in a variety of intracellular metabolic activities, such as the TCA, ETC, glycolysis, fatty acid β-oxidation and other processes (141, 142). For tumor cells, SIRT5 supports cell transformation and promotes breast carcinoma proliferation and tumorigenesis (143). Therefore, functional evaluation of SIRT5 should refer to its specific cell environment. Glutamine is converted into glutamate and ammonia in mitochondria by glutaminase (GLS), and glutamate is converted into α-ketoglutarate (α-KG) through glutamate dehydrogenase 1 (GLUD1) to participate in TCA (144). SIRT5 inhibits glutamine metabolism and regulates ammonia production by desuccinylation of GLS at Lys245 and/or Lys32, thus affecting autophagy (83). However, SIRT3 but not SIRT5 is co-immunoprecipitation with GLUD1 (83). After SIRT5 silencing or inhibition (by MC3482), autophagy is promoted, the expressions of MFN2 and OPA1 are decreased, and mitochondria are short-round shapes that cluster around the nucleus (83). In contrast, SIRT5 overexpression leads to the increase of MFN2 or OPA1, which is conducive to mitochondrial fusion, with increased number of mitochondria, elongated morphology, high ΔΨ(m), and reduced lactate production (83). STIR5, which regulates glutamine homeostasis, is also involved in mitochondrial dynamics regulation, suggesting there is a correlation between cellular metabolism and mitochondrial dynamics.

#### 3.2.9 MtSIRT for macrophage metabolism

The relationship between SIRT3/4/5 and macrophage metabolism has been emphasized in recent years. Generally speaking, SIRT3 promotes OXPHOS and FAO (fat acid oxidation), SIRT4 promotes glycolytic anabolism, and SIRT5 changes the metabolic state of macrophages by regulating PTM of key metabolic enzymes (100, 101, 145, 146). For example, in BMDM, SIRT3 deacetylates at Lys83 to activate PDHA1 (pyruvate dehydrogenase E1 α), then PDHA1 catalyzes the decarboxylation of pyruvate into acetyl-CoA and promotes OXPHOS, inhibits glycolysis, which helpful for reducing inflammation of macrophages (100). Interestingly, the ability of SIRT3 to promote OXPHOS and support FAO may lead to immune tolerance in monocytes (147). During the immune tolerance process, the increased SIRT4 controls the expression of PDK1 and SIRT1, thereby transforming the FAO pathway into glucose oxidation, promoting pyruvate to enter glycolysis, which is conducive to restoring immune homeostasis (145). The expression of SIRT4 is reduced in hepatocellular carcinoma (HCC) tumor tissues (101). By increasing the expression of lipid catabolisc genes, such as MCAD (medium chain acyl-CoA dehydrogenase), CPT1 (carnitine palmitoyl transferase 1), *etc.*, SIRT4 knockdown activates the FAO-PPARδ (peroxisome proliferator-activated receptor-δ)-STAT3 signal pathway and promotes the M2 markers (CD206, Arg-1) expression of TAM (101). This has also proved that SIRT4 is not conducive to FAO metabolic pathway from the opposite side. Moreover, SIRT4 silencing increases the expression of mitochondrial genes, including *PGC1α*, *EER-α*, *CytC*, *CoxV* (mitochondrial cytochrome oxidase V) and *IDH3α* (isocitrate dehydrogenase 3 alpha) in TAMs (101). PKM2 (M2-type pyruvate kinase), the physiological substrate of SIRT5, is a key determinant of the aerobic glycolytic transition of LPS-activated macrophages (102). In LPS (0.1 µg/mL)-stimulated BMDM, SIRT5-knockdown -regulated hypersuccinylation inhibits the pyruvate kinase activity of PKM2, promotes PKM2 dimerization entering into the nucleus, and ultimately mediates the up-regulation of IL-1β (102). Similarly, SIRT5 promotes inflammation of primary macrophages by promoting p65 acetylation (146). SIRT5 mediates the deglutarylation of GLUD1 in IL-4-polarized BMDM, thereby enhancing GLUD1 enzyme activity and promoting the production of αKG, which has a positive effect on the polarization of M2-like macrophages (148). It has also been shown that after the inhibition of SIRT5 by glutamine, PDH activity was enhanced due to the inhibition of PDHA1 desuccinylation, which promoted the polarization of IL-4 treated BMDM, and the glutamine metabolite α-KG played a role in the inhibition of SIRT5 (149).This seems to be the opposite of the previous sentence in which SIRT5 promotes α-KG production, possible causes include different disease models (eosinophilic chronic rhinosinusitis or burn sepsis) or different amounts of IL-4 stimulation (10 ng/mL or 50 ng/mL), but the specific reasons remain to be further explored (148, 149).

Therefore, mtSIRT is an important link between cellular metabolism and mitochondrial dynamics. SIRT3/4/5 promotes mitochondrial fusion by affecting OPA1 or/and MFN2, which produces different regulation on cell metabolism. SIRT3 promotes mitochondrial respiration, while SIRT4 and SIRT5 inhibit mitochondrial respiration in fibroblasts, MDA-ME-231 or C2C21. As for macrophages, SIRT3/4/5 affect macrophage metabolism in OXPHOS, glycolysis and FAO, illustrating that mtSIRT reconciles the metabolic state of macrophages to meet their demands. Unfortunately, these researches do not cover the change of mitochondrial dynamics under the remolding of macrophage metabolic state. Thence, there are complex regulatory mechanisms for mtSIRT to reconcile cellular metabolism and mitochondrial dynamics, and more researches are urgently needed to unearth their connection.

In conclusion, mitochondrial fusion is driven by multifactor and has undergone a biological process from OM fusion to IM fusion. In addition to mitochondrial fusion-related proteins regulating its morphology, ΔΨ(m), cristae formation, etc., some proteins have been confirmed to be involved in regulating macrophage metabolism (**Table 2**). However, there are few and scattered articles relating mitochondrial dynamics to macrophage metabolism, but we still conclude with a gratifying possibility. Whether there is a direct connection between mitochondrial fusion and macrophage metabolism and whether there is a logical relationship such as primary and secondary or causality is worthy of in-depth exploration.

## 4 Mitophagy and macrophage metabolism

Mitochondrial dynamics is involved in the elimination of part of damaged mitochondria and the maintenance of their own structural and functional integrity. When fusion/fission fails to meet the repair requirements, mitochondria are cleared by selective autophagy, also known as mitophagy. As researchers reviewed, the autophagosome mediated mitophagy is mainly induced by the LC3 adaptor protein, with or without ubiquitin-dependent or LC3 directly interacting with its receptor (150). In addition, some studies have pointed out that mitophagy requires Drp1 to break the mitochondrial network (151) or regulate PINK1/Parkin pathways (152, 153). The overexpression of OPA1 in myocardial infarction model induces mitophagy, and then increases the expression of antioxidant items (e.g., GSH, SOD), restores mitochondrial ΔΨ(m), blocks the accumulation of cytoplasmic cyt c, alleviates mitochondrial dysfunction, and ultimately saves cardiomyocytes from further damage (154). Both phenomena remind us that mitochondrial dynamics possibly participates in the regulation of mitophagy. However, the internal relationship between mitochondrial dynamics and mitochondrial autophagy remains unclear. Meaningfully, Fun-14 domain containing protein 1 (FUNDC1), a key receptor associated with mitochondrial fission and mitophagy, was discovered. OPA1 and DNM1L (DRP1 Dynamin 1-like) are core targets of FUNDC1 (155). In the process of hypoxia-induced mitophagy, FUNDC1 first acts on the MAMs and is enriched at the mitochondrial associated membrane (MAM), and then recruits DNM1L/DRP1 and separates from CANX to promote mitochondria fission in response to hypoxic stress (61). When Lys70 (K70) of FUNDC1 is mutated to Ala (A) [not Arg (R)], the interaction of FUNDC1 on OPA1 reduced and that of DNM1L is enhanced, which in turn stimulates mitophagy (155). However, recent studies have found that mitophagy caused by FUNDC1 protects the kidney from ischemic damage by inhibiting DRP1-induced mitochondrial fission (62). Therefore, the effect of FUNDC1 on mitochondrial fission depends on the microenvironment of cells, illustrating the metabolic state of cells may alter mitochondrial dynamics. In addition, other connections between mitochondrial dynamics and mitophagy deserve further investigation. On the other hand, FUNDC1-deficient mice experienced increased liver damage, accelerated adipose tissue macrophage infiltration and M1 polarization, and were accompanied by metabolic changes such as up-regulation of genes associated with adipogenesis (156). Overexpression of FUNDC1 inhibited the production of IL-1β and ROS in BMDMs (157). Therefore, FUNDC1-mediated mitochondrial autophagy has an effect on the reprogramming of macrophage metabolic state. However, the existing body of research predominantly focuses on FUNDC1’s canonical role in mitophagy regulation during disease pathogenesis, wherein macrophage metabolic parameters are occasionally measured as secondary endpoints. This conspicuous gap in direct mechanistic investigations positions the FUNDC1-macrophage metabolism axis as a novel and scientifically significant area of investigation.

Choline transporter 1 (CTL1) -mediated choline uptake affects mitochondrial phosphatidylcholine (PC) and sphingomyelin (SM) (158). Impaired choline uptake disrupts mitochondrial ATP synthesis and triggers AMPK activation to promote DRP1-driven mitophagy (158). Moreover, LPS (0.2 µg/mL) enhances the expression of CTL1 for 4h, and then promotes the choline uptake of BMDM (158). After 24h of LPS treatment, the lipid metabolism of macrophages (mainly occurring in mitochondria) is remodeled, for example, mitochondrial PC (mito-PC) decreased and mito-SM increased (158). In conclusion, it is reasonable to assume that choline acts as a connecting factor between mitophagy and macrophage metabolism. Besides, choline is considered to be a water-soluble vitamin related to the B vitamins, which is involved in the synthesis of acetylcholine and the formation of methyl donors in the methionine cycle in the body (159). Whether the above-mentioned regulation of mitochondrial dynamics and macrophage metabolism by choline is directly regulated or occurs through these biosynthetic or oxidative processes is worth exploring at this point (**Figure 2**).

## 5 The connection of macrophage metabolism with “kiss and run”

In addition to the above-mentioned mitochondrial complete fission and fusion, mitochondria have a transient fusion-fission mode called “kiss and run”. This process can be completed in a very short time (as short as 4 seconds), and relative to the longitudinal alignment of fully fused mitochondria, transient fusions tend to occur from oblique or lateral sites, so transient fusions always preserve the original topology (160). During transient fusion, the soluble contents of mitochondria undergo passive diffusion under the MFNs- and OPA1-dependent action (160). Transient fusion occurs in mitochondria on two microtubules, and DRP1 is recruited when transient fusion occurs due to the force of motor proteins and the tension of anchoring to the microtubules, causing transient fission (160). Liu et al. also found that transiently fused mitochondria maintain normal bioenergetics such as ΔΨ(m) and respiration (160). To sum up, the “kiss and run” mode is similar to that of athletes on two tracks. After a brief high-five, they exchange part of the material, and then quickly separate without affecting each other’s motivation.

The instantaneity of mitochondrial kiss and run enables it to exchange or dilute part of its contents in the shortest time without affecting mitochondrial metabolism. Although the role of kiss and run in macrophage metabolism is currently lacking, we believe this will be a meaningful research direction.

## 6 Metabolism-related diseases of macrophages are linked to mitochondrial dynamics

### 6.1 Atherosclerosis

Macrophage metabolic reprogramming determines the progression of atherosclerosis. During the transition from early to inflammatory progression of atherosclerosis, the proportion of M[LPS+IFN-γ]-like macrophages gradually increased, accompanied by the increase in glycolysis, and in the regression stage of atherosclerotic plaques, the proportion of M[IL-4]-like macrophages increased with up-regulated FAO, and M(Ox)-like macrophages with lower phagocytic migration ability are also found (161).

In the dynamic regulation of mitochondria, mitophagy removes damaged mitochondria and plays a role in preventing oxidative stress and apoptosis (150), so mitophagy is crucial for cardiovascular-derived cellular homeostasis (162). Studies have linked impaired mitophagy to atherosclerosis (163). For example, treatment with the DRP1 inhibitor mdivi-1 reduces mitochondrial fragmentation and attenuates atherosclerosis in diabetic ApoE^-/-^ mice (164). This is a very interesting point. Although the metabolic state of macrophages in an atherosclerotic environment is variable, we can treat atherosclerosis by regulating mitochondrial dynamic to active mitophagy as an entry point.

### 6.2 Asthma

Asthma is a chronic inflammatory disease of the respiratory system, which is divided into various phenotypes according to the immune cells enriched in the airways (165). Macrophage function is altered in asthmatic patients, including decreased phagocytic capacity, increased production of inflammatory mediators, and disturbed production of anti-inflammatory factors and so on, moreover, there is substantial evidence that macrophages may serve as therapeutic targets for asthma (165).

Metabolic alterations in human and mouse airway cells under cigarette smoke (CS) suggest a vital role for mitochondria, specifically, the mitochondrial TCA and OXPHOS are converted to FAO, and mtROS increases with increasing damage, and the ATP demand for cellular repair increases at this time (166). In chronic obstructive pulmonary disease (COPD), studies have found increased mitophagy in airway epithelial cells, and unfortunately, no studies have linked mitochondrial dynamics to macrophage therapy for asthma (167). We speculate, however, that the metabolic reprogramming of macrophages by regulating mitochondrial dynamics would be a possible strategy for the treatment of asthma. Emerging mechanistic studies suggest that DRP1 is a key amplifier in multifactorial asthma, establishing its therapeutic potential through pathway regulation (168–170). Notably, in recombinant DEK-induced mouse asthma models, DRP1 expression is significantly upregulated and physically interacts with ATAD3A (169). Mechanistic inquiry revealed that inhibition of ATAD3A mediates inhibition of DRP1, thereby resolving mitochondrial oxidative stress by regulating mitophagy, and achieving attenuation of airway inflammation (169). In ovalbumin-induced asthma, mitochondrial fusion/fission dysregulation promotes ROS production and intensifies the activation of NLRP3 inflammasome (171). After Abscisic acid treatment, NLRP3 activation was down-regulated, mitochondrial fusion/fission markers such as OPA1, MFN2 and DRP1 were decreased, and the expression of PPARγ was further increased, which inhibited airway inflammation (171). Studies have shown that PPARγ regulates FAO metabolism in macrophages (172). Therefore, mitochondrial dynamics and metabolic adaptation are key regulators of macrophage behavior in asthma.

### 6.3 Other diseases

A recent article summarizes the relationship between mitophagy and macrophage-related chronic inflammatory and autoimmune diseases (173), including inflammatory bowel disease (IBD), systemic lupus erythematosus (SLE), and primary biliary cirrhosis (PBC). Although this article does not mention the role of mitochondrial dynamics in IBD, SLE, and PBC, according to our previous description of mitochondrial dynamics perturbation and mitophagy, we believe that mitochondrial dynamics regulation may be related to macrophages metabolic reprogramming through disturbing mitophagy ultimately serves as a target for the treatment of macrophage-related diseases.

## 7 Conclusion

Mitochondria are the factories of cell energy metabolism and the key organelles to maintain body homeostasis (174). With the change of cell microenvironment, mitochondria undergo a series of dynamic changes, and form a complex regulatory network through hyperfusion, elongation, fragmentation, etc. (18, 20), to adapt to or meet the needs of cell metabolism and immunity. When the dynamics of mitochondrial fission and fusion is unable to rescue the cell, mitophagy occurs (150), thereby eliminating the damaged mitochondria. Recently, researchers corrected some statements about mitochondrial morphology, that is the hyperfused and fragmented mitochondrial network are caused by the equilibrium between fission and fusion processes, and thus affect mitochondrial functional fitness, rather than described mitochondrial morphology as single fusion and/or fission (20). The up-regulated fusion to fission ratio promotes mitochondrial hyperfusion whether fusion increases or fission decreases, or both, instead, when the ratio of fusion to fission decreased, mitochondria tend to fragmentation. Intrinsically, the equilibrium fluctuations described above are more consistent with natural phenomena, but in order to describe mitochondria in different states more intuitively, fusion and fission are also used to describe the morphology of mitochondria in this paper.

Macrophages are important members of the natural immunity, and their polarization is accompanied by glycolysis and OXPHOS metabolic programming (85). These metabolic changes are inseparable from the regulation of mitochondria. Therefore, previous studies focused on the association between mitochondrial metabolism and macrophage immunity, such as the dual role of ERK1/2 on mitochondrial fission (35, 175, 176), and the role of PKA and AKT on mitochondrial fusion (20).

Based on existing literature, we reviewed the dynamic changes of mitochondrial dynamics during macrophages polarization and the effects of mitochondrial dynamics on the functional diversity of macrophages. In LPS-activated macrophages (M1 type or classically activated), enhanced phosphorylation of DRP1 at Ser616 promotes mitochondrial fission, reduced ΔΨ(m), and morphological changes such as mitochondrial fragmentation and disorganized cristae (22). During this process, the remodeled mitochondria redirect their function towards increased ROS production, thereby driving the transcription of inflammatory cytokines and exacerbating the inflammatory response (22). On the other hand, mitochondrial fission acts as an atypical regulator of macrophage functional plasticity. For example, DRP1-mediated mitochondrial fission is required for the uptake of apoptotic cells by macrophages (177). Mitochondrial fission promotes Ca^2+^ release from ER, activates vesicular trafficking and cytoskeletal remodeling, enabling macrophages to sequentially engulf multiple apoptotic cells without functional exhaustion (177). Importantly, mitochondrial fission and increased cytoplasmic calcium prompt protein kinase C-θ of macrophages to phosphorylate WIP (Wiskott-Aldrich Syndrome Interacting Protein) during phagocytosis and are necessary for therapeutic antibody-induced phagocytosis of live tumor cells (178). Furthermore, by modulating metabolic pathways through the inhibition of Glutamine-fructose-6-phosphate transaminase 2 (GFPT2) in tumor cells, glutamine metabolism is regulated, thereby restoring mitochondrial dynamics and consequently influencing the phagocytic capacity of macrophages (178). Mdivi-1 inhibits DRP1-dependent mitochondrial fission, reduces mitochondrial reactive oxygen species (mito-ROS) and NLRP3 inflammasome activation, thereby reducing macrophage M1 polarization and down-regulating IL-6 and TNF-α release, which is beneficial for alleviating atherosclerotic diseases (179). Importantly, it was found that following M1 polarization, the mitochondrial ETC complex of macrophages was either partially or completely suppressed, leading to impaired OXPHOS function, and macrophages were unable to transform to M2 phenotype even when exposure to IL-4 stimulation (180). However, macrophages showed a tendency to increase M2 markers (e.g., CD206 and IL-10) after DRP1 inhibition by Mdivi-1 (179), suggesting that mitochondrial dynamics may acts as metabolic rheostats, allowing macrophages to switch between polarized states. Alternatively activated macrophages (M2-like) are predisposed to mitochondrial fusion, produce elongated mitochondria, and have high levels of FAO and OXPHOS (21). As previously discussed, OPA1, MFN1/2, and other proteins participate in the dynamic regulation of mitochondrial fusion (39, 125). Research has shown that restoration of mitochondrial dynamics by up-regulation of MFN1/2, which promotes mitochondrial fusion, successfully inhibits pro-inflammatory macrophage polarization (181). The fused mitochondria facilitate FAO and ATP synthesis, presenting an optimal therapeutic strategy to promote M2 polarization in chronic inflammatory conditions (181). However, the effect of mitochondrial fusion-associated proteins on macrophage function appears to be more complex than a simple M2-to-M1-type conversion. Myeloid specific OPA1 deletion in mice leads to mitochondrial network fragmentation and cristae disorganization (182). Interestingly, macrophages deficient in OPA1 exhibit up-regulated expression of M2 markers such as Arg1, Mrc1 and Retnla, suggesting that OPA1 deletion promotes M2-like phenotype (182). Paradoxically, under M2-polarized conditions, OPA1 deletion also interferes with macrophage metabolism and immune pathways by shifting macrophages to glycolytic metabolism and inability to maintain ATP levels due to mitochondrial uncoupling (182). OPA1 deletion also disrupts TCA cycle, resulting in succinate and citrate accumulation and metabolic perturbation, which then impairs NF-κB/p65 signaling and ultimately suppress the pro-inflammatory response (182).

Regulation of mitochondrial dynamics-associated proteins, including DRP1 and OPA1, reshaped the metabolic profile of macrophages (179, 182). DRP1-mediated mitochondrial fission disrupts cristae morphology and enhances mito-ROS generation (179). Elevated mito-ROS stabilizes hypoxia-inducible factor-1α (HIF-1α), driving a metabolic shift toward glycolysis characterized by increased glycolytic ATP production and enhanced glycolytic flux (183). OPA1 deficiency induces macrophage dependency on glycolysis; however, defective assembly of respiratory chain supercomplexes and impaired ATP production compromise the acquisition of the M1 phenotype, leading instead to the manifestation of classical M2 characteristics (182). Enhancing OPA1 activity or inhibiting its degradation restores OXPHOS capacity (184). Thus, OPA1 functions as a “metabolic switch” in macrophage polarization by orchestrating mitochondrial dynamics and metabolic homeostasis. Consistently, we summarize and induction that mitochondrial dynamics are closely related to macrophage metabolism. A large number of mitochondrial fission/fusion-related proteins have been shown to affect macrophage metabolism. Among them, FAM73b (26), MTCH2 (84), SLP-2 (79), and mtSIRT (80, 82, 83, 100–102) related to mitochondrial fusion, Fis1 and MTP18 related to mitochondrial fission are closely related to macrophage metabolism. Specifically, mitochondrial fusion tends to macrophage OXPHOS, while mitochondrial fission tends to glycolysis and fat acid metabolism. These proteins serve as the vinculum between mitochondrial dynamics and macrophage metabolism. PTM plays an important role in mitochondrial dynamics and macrophage metabolism, especially in DRP1 (22, 41) and mtSIRT (100–102). MiRs are involved in the regulation of macrophage polarization (185) and mediate metabolic shifts (186). Although studies on the regulation of macrophage metabolism by miRs are lacking, we suggest that miRs may be the link through which mitochondrial dynamics regulate macrophage metabolism (82, 84, 96).

In addition, we have conducted a comprehensive search for the relationship between mitophagy (150, 154, 158, 159), mitochondrial “kiss and run” (160) and macrophage metabolism. Although relevant research is insufficient, it still needs to be verified. Mitocytosis, a recently discovered new mitochondrial quality control mechanism, has the ability to maintain mitochondrial homeostasis in macrophages (187). As mitocytosis is a newly discovered mechanism, its impact on macrophage metabolism is currently unknown, but it would be an interesting research direction. Study of Chen et al. revealed that the antimalarial drug chloroquine (CQ), as an anti-tumor immunomodulator, transformed TAM from M2 to tumor killing M1 phenotype (transformed TAM to tumor-killing macrophages) by reprogramming the transformation mechanism of TAM metabolism from OXPHOS to glycolysis (188), which was a strategy to improve the adaptability of macrophages in immunosuppressed environment. This suggests that mitochondrial dynamic regulation has huge potential to be used as a means of metabolic reprogramming of macrophages to enhance the resistance of macrophages in adverse environments such as immunosuppression, thus contributing to host survival. On the other hand, we summarize the relationship between macrophage-related chronic inflammatory diseases and autoimmune diseases (including atherosclerosis, asthma) and mitochondrial dynamics, and further suggest that mitochondrial metabolism regulation may be an effective strategy for the treatment of these diseases. Overall, the relationship between mitochondrial dynamics and macrophage metabolism still requires a large amount of experimental input to verify its logical relationship.

## Glossary

AAAC: ADP/ATP carrier
AKT: Protein kinase B
AML: acute myelocytic leukemia
AMP: Adenosine 5’-monophosphate
AMPK: AMP-activated protein kinase
ARL2: ADP-ribosylation factor (ARF) like 2
Arp2/3: Actin-related protein 2/3
BAT: Brown adipose tissue
BMDM: Bone marrow derived macrophages
BMSCs: Bone marrow mesenchymal stem cells
cAMP: Cyclic adenosine monophosphate
CANX: Calnexin
CL: Cardiolipin
COPD: Chronic obstructive pulmonary disease
CoxV: Mitochondrial cytochrome oxidase V
CPT1: Carnitine palmitoyl transferase 1
CQ: Chloroquine
CS: Cigarette smoke
CTL1: Choline transporter 1
Cyt c: Cytochrome c
DAG: Diacylglycerol
DNM1L: DRP1 Dynamin 1-like
DRP1: Dynamin-related protein 1
ELMOD2: ELMO domain containing 2
ER: Endoplasmic reticulum
ERK: Extracellular signal-regulated kinase
ESCs: Embryonic stem cells
ETC: Electron transport chain
ETS: Electron transport system
FAO: Fat acid oxidation
Fis1: Mitochondrial fission 1 protein
FUNDC1: Fun-14 domain containing protein 1
Fzo: fuzzy onions
GABA: γ-aminobutyric acid
GAP: GTPase-activating protein
GFPT2: Glutamine-fructose-6-phosphate transaminase 2
GLS: Glutaminase
GLUD1: Glutamate dehydrogenase 1
GSH: Glutathione
GTPase: Guanosine triphosphatases
HCC: Hepatocellular carcinoma
IBD: Inflammatory bowel disease
IDH3α: Isocitrate dehydrogenase 3 alpha
IFN-β: Type I interferons
IM: The inner membrane
IMM: Mitochondrial inner membrane
IMS: The intermembrane space
INF2: Inverted formin 2
IRF1: Interferon regulator 1
JAK: Janus kinase
L-OPA1: Long-OPA1
MAM: Mitochondrial associated membrane
MCAD: Medium chain acyl-CoA dehydrogenase
MDA: Malondialdehyde
MEFs: Mouse embryonic fibroblasts
Mff: Mitochondrial fission factor
MFN1/2: Mitofusin 1/2
MiD: N-terminally anchored mitochondrial dynamics proteins
Miga: Mitoguardin
MIMP: Met-induced mitochondrial protein
MiR: MicroRNA
Mito-PC: Mitochondrial PC
MitoPLD: Mitochondrial phospholipase D
Mito-ROS: Mitochondrial reactive oxygen species
Mito-SM: Mitochondrial SM
MOMP: OMM permeabilization
MSTO1: Misato homolog 1
MTCH2: Mitochondrial carrier homolog 2
mtDNA: Mitochondrial DNA
MTP18: Mitochondrial protein 18 kDa
MtSIRT: Mitochondrial sirtuins
NF-κB: nuclear factor kappa-B
OGA: O-GlcNAcylation
OM: The outer membrane
OMM: Mitochondrial outer membrane
OPA1: Optic atrophy 1
OXPHOS: Oxidative phosphorylation
PA: Phospholipid acid
PBC: Primary biliary cirrhosis
PC: Phosphatidylcholine
PDH: Pyruvate dehydrogenase
PDHA1: Pyruvate dehydrogenase E1 α
PGC1α: PPARγ co-activator 1 alpha
PKA: Protein kinase A
PKM2: M2-type pyruvate kinase
PLD: Phospholipase D
PPAR-γ: peroxisome proliferator-activated receptor-γ
PPARδ: Peroxisome proliferator-activated receptor-δ
PTM: Post-translational modification
RIP1: receptor-interacting protein kinase 1
SAM: S-adenosylmethionine
SLE: Systemic lupus erythematosus
SLP-2: Stomatin-like protein 2
SM: Sphingomyelin
SOD: Superoxide dismutase
S-OPA1: Short-OPA1
Spire1C: Spiretype actin nucleation factor 1
STAT: signal transducer and activator of transcription
Stat2: Signal transducers and activators of transcription 2
TAM: Tumor-associated macrophage
tBID: Truncated BID
WAT: White adipose tissue
WT: Wild type
α-KG: α-ketoglutarate
ΔΨ(m): Membrane potential
